# Tuning the charge flow between Marcus regimes in an organic thin-film device

**DOI:** 10.1038/s41467-019-10114-2

**Published:** 2019-05-07

**Authors:** A. Atxabal, T. Arnold, S. Parui, S. Hutsch, E. Zuccatti, R. Llopis, M. Cinchetti, F. Casanova, F. Ortmann, L. E. Hueso

**Affiliations:** 1 CIC nanoGUNE, 20018 Donostia-San Sebastian, Basque Country, Spain; 2 Center for Advancing Electronics Dresden and Dresden Center for Computational Materials Science, Technische Universität Dresden, 01062 Dresden, Germany; 3 Experimentelle Physik VI, Technische Universität Dortmund, Dortmund, 44221 Germany; 4 IKERBASQUE, Basque Foundation for Science, 48013 Bilbao, Basque Country, Spain; 5 Simbeyond B. V., 5612 AE Eindhoven, The Netherlands; 6 IMEC, Kapeldreef 75, 3001 Leuven, Belgium; 7 K. U. Leuven, Arenbergpark 10, 3001 Leuven, Belgium

**Keywords:** Electron transfer, Electronic devices, Molecular electronics

## Abstract

Marcus’s theory of electron transfer, initially formulated six decades ago for redox reactions in solution, is now of great importance for very diverse scientific communities. The molecular scale tunability of electronic properties renders organic semiconductor materials in principle an ideal platform to test this theory. However, the demonstration of charge transfer in different Marcus regions requires a precise control over the driving force acting on the charge carriers. Here, we make use of a three-terminal hot-electron molecular transistor, which lets us access unconventional transport regimes. Thanks to the control of the injection energy of hot carriers in the molecular thin film we induce an effective negative differential resistance state that is a direct consequence of the Marcus Inverted Region.

## Abstract (web summary)

To demonstrate charge transfer in different Marcus regimes in an organic semiconductor, precise tuning of the material’s electronic properties is required. Here, the authors use a three-terminal hot-electron technique to access the Marcus regimes for electronic transport in organic thin films.

## Introduction

Electronic transport in molecular solids is commonly described by a hopping mechanism, in which the carriers are capable of moving from one molecular site to another thanks to both the thermal energy that leads to their activation and the driving electric field^1–10^. This behaviour contrasts with the transport mechanism observed in other solids, such as metals or some inorganic semiconductors with relatively wide transport bands in which the electronic carriers can flow freely between scattering events^11,12^. In spite of the hopping transport being a sequence of cumbersome individual electron transfer events, electronic conduction in molecular materials enables a wide range of electronic and opto-electronic applications and it is crucial in devices such as organic field-effect transistors^13,14^, organic light-emitting diodes^15,16^ and organic photo-voltaic cells^17,18^.

Marcus presented several decades ago a pioneering theoretical proposal regarding the incoherent electron transfer events between redox partners such as molecules, which would be applicable in the first instance to chemical species dissolved in a solution^19,20^. In a series of papers, he described the influence of molecular and environmental conformations and the energetic driving force for the electron transfer rates. The theory culminated in the prediction of an unconventional transport regime termed Marcus Inverted Region (MIR), in which an increase in the driving force leads to a reduction in the transfer rate^19–22^. Although Marcus’s theory in its later quantum formulation is of very general applicability and great popularity, it has been difficult to verify it experimentally in molecular solids. Moving toward that target, Yuan et al.^23^ have recently discussed MIR in a molecular junction based on a self-assembled monolayer and intramolecular orbital gating which relies on the charging of the molecules. In general terms, to be able to address this unconventional transport regime requires an active control over the driving force for the charge carriers, which, for instance, could be provided by differences in their electronic energies. As a result of tuning the involved energies, the crossover point between the MIR and the normal region should exhibit a minimum in the activation energy for transport (see below). Here, we propose an alternative path to that of Yuan et al. who realized a model of Migliore et al.^10,24^ predicting a maximum in the activation energy. This allows us to demonstrate experimentally and computationally a MIR in organic thin films that are similar to those employed in various opto-electronic applications.

More specifically, by making use of the injection of hot-electrons in a solid-state device, we can explore the Marcus transport regimes in fullerene-based thin film devices. Our experimental data show a tuneable effective negative differential resistance (NDR)^25–28^ arising from the Marcus inversion phenomenon^19^. Our theoretical considerations and comprehensive simulations that take into account the complex transport phenomena in the full device are able to reproduce and rationalize these data in detail. Building on this fundamental achievement, the observation and control of the effective NDR in a three-terminal transistor opens the way to the engineering of molecular electronic amplifiers and effectively lossless oscillators^27,29^.

## Results

### Experimental device

**Fig. 1:**
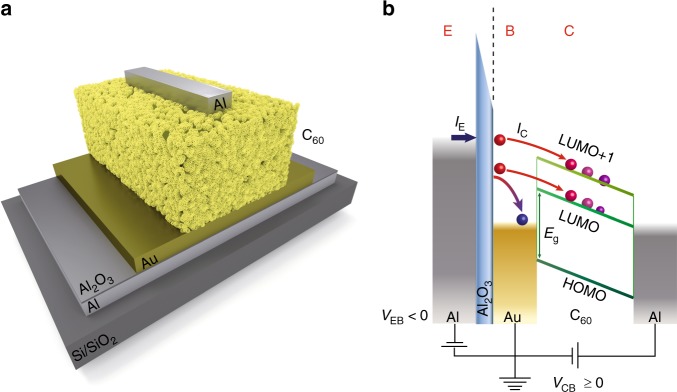
Device schematics. **a** Schematic cross section of the device. **b** Hot-electron transistor operation. Electrons are injected by applying a negative emitter-base bias, *V*_EB  _< 0 V, and detected in the molecular semiconductor. These electrons are out of equilibrium with the thermal electrons in the base which cannot be described by a larger temperature. The measurements can be performed either without any externally applied collector-base bias, *V*_CB_, or by *V*_CB _ ≥ 0 V

Our experiments are performed in a three-terminal vertical device, which is composed of an aluminium/aluminium oxide emitter (E), a metallic base (B) and a semiconductor collector with an aluminium top contact (C) (see Fig. 1)^30,31^. In this work, as a proof of principle, gold (Au) as base and both n-type C_60_ and C_70_ fullerene as collector have been used (see Fig. 1a). Details on the device fabrication and thin film characterization can be found in the Methods section [atomic force microscopy and x-ray diffraction measurements are shown in Supplementary Figs. 1, 2, respectively]. Figure 1b shows the working principles of the hot-electron transistor. When a negative bias *V*_EB_ is applied, a current *I*_E_ is injected from the emitter into the base by tunnelling through the Al_2_O_3_ barrier. These electrons are “hot” in the base as their energy is well above ($\gg$*k*_B_*T*) the Fermi level of the metal, and a small fraction of them crosses the thin metallic base ballistically without any significant energy attenuation^30–32^. For the case in which the applied bias −*eV*_EB_ ≥ Δ (Δ is the metal-semiconductor energy barrier), some of the injected hot electrons enter into the C_60_ LUMO states, while the remaining ones flow back into the base. At higher energies, electrons can also enter higher energy conductive states of C_60_, such as LUMO + 1. Since C_60_ is an n-type semiconductor and is sandwiched between two metallic contacts with different work functions, the existing intrinsic built-in potential enables the detection of the electron current, *I*_C_, without any applied collector-base bias, *V*_CB_ or with a positive *V*_CB_^33,34^.

### Temperature dependence of C_60_ and C_70_ in-device molecular spectroscopy (i-MOS)

**Fig. 2:**
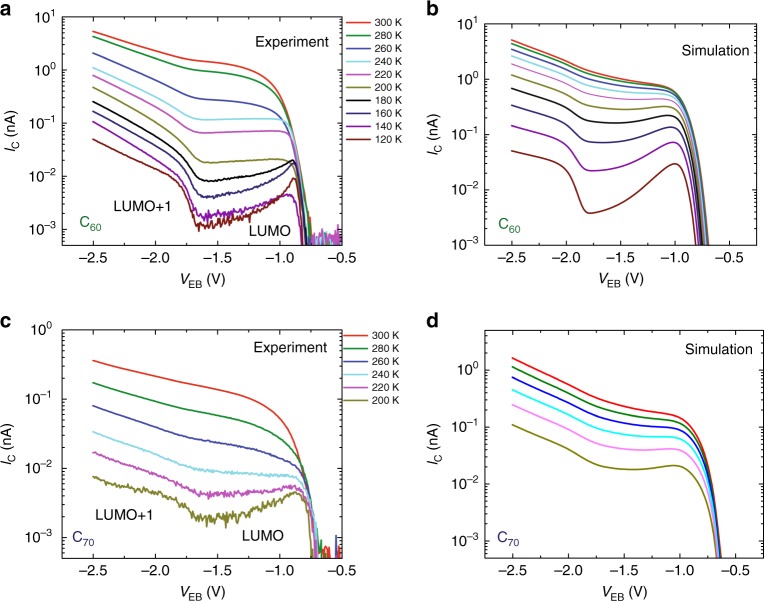
Temperature dependence of the hot-electron current. **a** Temperature dependence of the direct hot-electron current *I*_C_ of a Au/C_60_ based in-device molecular spectroscopy (i-MOS) device for negative emitter-base bias *V*_EB_ < 0 V, and without any applied collector-base bias, *V*_CB_ = 0 V. **b** Simulated temperature dependence of *I*_C_ for the Au/C_60_ device at *V*_CB_ = 0 V. **c** Temperature dependence of *I*_C_ of a Au/C_70_ based device for negative emitter-base bias *V*_EB_ < 0 V, and without any external bias *V*_CB_ = 0 V. **d** Simulated temperature dependence of *I*_C_ for the Au/C_70_ device at *V*_CB_ = 0 V

Figure 2a shows the temperature dependence of *I*_C_ with *V*_EB_ < 0 V and *V*_CB_ = 0 V from 300 K to 120 K in a C_60_-based device [see Supplementary Fig. 3 for individual plots in linear scale]. The peaks at *V*_EB_ = −0.8 ± 0.1 V and *V*_EB_ = −1.7 ± 0.1 V correspond to LUMO and LUMO + 1, respectively. The increase of *I*_C_ between LUMO and LUMO + 1 at high temperatures is observed to converge into a plateau at temperatures around 240 K. At lower temperatures, below 180 K, an effective NDR is observed between LUMO and LUMO + 1. This is a truly unconventional behaviour: in a simple picture when more electrons are provided, the current should always increase with the driving voltage since we are collecting all the hot electrons injected into the molecular material. The same behaviour has been observed for C_70_-based hot-electron devices (Fig. 2c), with the NDR observable below 220 K. In the case of C_70_, the peak at *V*_EB_ = −0.7 ± 0.1 V corresponds to the LUMO and the one at *V*_EB_ = −1.6 ± 0.1 V to LUMO + 1. The measurements of C_70_ devices [see Supplementary Fig. 4 for individual plots in linear scale] are performed until 200 K due to the lower *I*_C_ current compared to C_60_, which is a consequence of the lower intrinsic mobility of the former molecular material.

### Marcus Inverted Region

In order to rationalise this intriguing regime, a comprehensive simulation framework was employed which allows us to understand the device transport in detail (Fig. 2b, d). It is based on simulations under the same conditions as in the experiments (for details see Supplementary Note 1). Charge transport across the interface and throughout the film is modelled with a Fermi’s golden rule expression for the transfer rate. Such an approach is justified for the weak electronic coupling between the involved states (e.g., delocalized metal states and localized molecular orbitals, see Supplementary Note 1 and Supplementary Fig. 5 for further theoretical considerations).

Taking into account conformational changes of the molecules in the electron transport yields an expression known as Marcus hopping rate^22^

1 $$k \propto {\mathrm{exp}}\left[ { - \frac{{\left( {\Delta G - \Lambda } \right)^2}}{{4k_BT\Lambda }}} \right]$$

where $\Lambda$ is the reduced reorganization energy^35^ of the charge-transfer event, $\Delta G$ the difference in the energies of initial and final states and *k*_*B*_*T* the thermal energy.

**Fig. 3:**
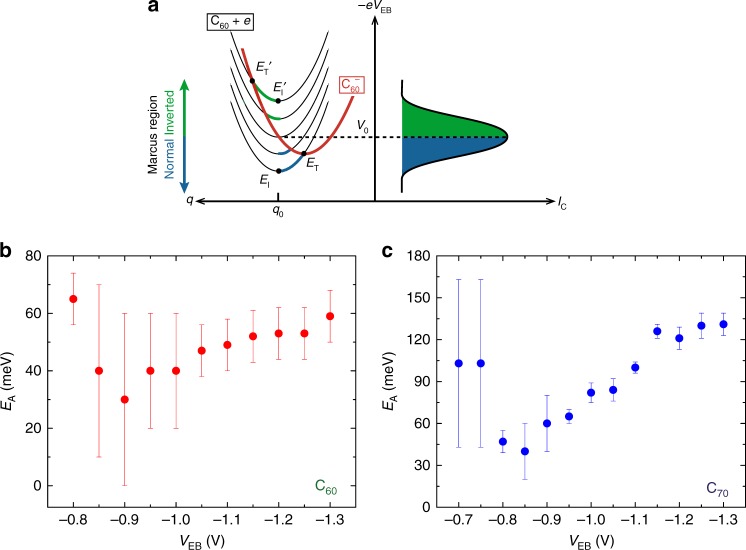
Marcus Inverted Regime and activation energy. **a** Illustration of the charge transport in both the Marcus Normal and Inverted Regions. The electronic energy of a neutral C_60_ (to whom the hot electrons have not yet reached) plus the hot-electron energy are shown on the left as black parabolas, which are vertically offset by different emitter-base voltages (which correspond to the variation in hot-electron energy). The parabolic shape indicates the dependence of the energy on the deformation along a molecular conformation coordinate, *q*. The analogous dependence for the negatively charged ${\mathrm{C}}_{60}^ -$ (in its final configuration, having received an electron) is shown in red. The crossing points between the parabolas define the transition energy for the charge transport, which is controlled by the emitter-base voltage. As an example, two cases are indicated with transition states *E*_T_ and *E*_T_’, which are activated from initial states *E*_I_ and *E*_I_’, respectively. The activation energy is defined in general as the difference between the transition and the initial energies such that *E*_A_ = |*E*_T_ − *E*_I_|. At low voltages, exemplified in the transition from *E*_I_ to *E*_T_, the barrier (left) decreases and the output current (blue shaded area on the right) increases as the voltage increases in which is commonly denoted as Marcus Normal Region. The barrier is minimised at *V*_0_ leading to the maximum injection current (right). For an even larger driving force, exemplified now in the transition from *E*_I_’ to *E*_T_’, the current decreases (green shaded area on the right) due to the increased barrier. This is commonly called the Marcus Inverted Region. **b** Dependence of the activation energy *E*_A_ of C_60_ on *V*_EB_, which shows a minimum at the *V*_EB_ = −0.9 V position. Error bars are from fit uncertainties (s.e.m.). **c** Dependence of the activation energy *E*_A_ of C_70_ on *V*_EB_, which shows a minimum at the *V*_EB  _= −0.85 V position. Error bars are fit uncertainties (s.e.m.)

A key point in the Marcus theory is the thermal activation of charge transfer, which is reflected in Eq. (1) by the temperature dependence of the negative exponent. It can be further described by an activation energy *E*_A_, which is defined as the difference between the energy of the initial state *E*_I_, and the energy of the transition state *E*_T_ (energies are indicated in Fig. 3a). In our hot-electron transistors, due to its unique working principle, we can choose the former by tuning the hot electron energy by *V*_EB,_ which shifts the initial state parabola relative to the final state parabola and manipulates directly *E*_I_. In other words, *V*_EB_ acts as gating in our transistor configuration. This setup is different from the situation considered by other models in which a conventional gate voltage is applied to the molecules^24^. In our case, Fig. 1b illustrates how this knob allows increasing the driving force for electron transport by tuning *V*_EB_ around the LUMO position. In this way, at low *E*_I_ ($- V_{{\mathrm{EB}}} \, < \, V_0$), the voltage increase results in a lower activation energy and leads to an increased current (illustrated in blue in Fig. 3a). This behaviour is known as the Marcus Normal Region. However, the non-monotonous dependence of the activation energy on *V*_EB_ establishes another regime, which is the so-called MIR. This regime is characterised by the reduction of the current despite a continuous increase in the driving force (larger *E*_I_). At the voltage −*V*_EB_ = *V*_0_ (at which *E*_I_ = *E*_T_), the activation energy takes a minimum and a crossover between the normal and inverted Marcus regions occurs. The two regimes can be observed both in the experimental data and in the corresponding simulations shown in Fig. 2.

We note that Migloire, Schiff and Nitzan^24^ presented previously a model for transport in metal-molecule-metal junctions in which electrons from the leads are thermal in the sense that they are described by Fermi distributions and chemical potentials and do not consider hot electrons. While the model potentially includes electrons in the MIR, in their considered transport geometry the resulting current through the biased junction would be dominated by electrons injected from below the Fermi level (non-MIR electrons), because of the higher transfer rate of those non-activated electrons from the leads. As opposed to that situation, in our system the hot electrons are the responsible for the current in the different Marcus regions and the thermal current is negligible, thus the previous model is not directly applicable. In Supplementary Fig. 6, we present additional simulations without the hot-electron distribution from the tunnel barrier in our device (akin to metal-molecular layer-metal junctions) in which we confirm the absence of the NDR, thus confirming the relevance of hot electrons.

The *V*_EB_ dependence of the activation energy, *E*_A_, coming from an Arrhenius analysis of the temperature dependence of the transport of C_60_ and C_70_ are shown in Fig. 3b, c, respectively. A reduction in *E*_A_ is observed with the minimum at *V*_EB  _= −0.9 V for the case of C_60_ and at *V*_EB  _= −0.85 V for the case of C_70_, which corresponds to the *V*_0_ level in the Fig. 3a. In these figures one can observe how the *E*_A_ is progressively reduced until a minimum point after which it progressively increases. The region in which *E*_A_ decreases corresponds to the Normal Marcus region, while the one where *E*_A_ increases corresponds to the MIR (see Supplementary Fig. 7 and Supplementary Table 1). Besides this direct evidence at low temperatures, our simulations can also explain the reduction of the NDR in the hot-electron current measurements at high temperatures (Fig. 2a, c) taking into account the increase of the inelastic scattering in the base with temperature (see Fig. 2b, d, Supplementary Table 2 and Supplementary Note 1)^36^. In addition, Supplementary Fig. 5 provides further insights into how a monotonous standard hopping rate fails to reproduce our results, while Supplementary Fig. 8 shows that the observation of NDR does not depend on the thickness of the molecular layer in our thin film regime.

The observation of this captivating energy crossover in the context of analysing redox reactions in solution chemistry marked the breakthrough of Marcus theory^21^. While in the original context this required a number of experiments with a series of redox partners^37^, interestingly in the present case, we demonstrate it for each of the compounds individually, i.e., they remain unchanged during the whole measurements. Here we are able to directly access this transport regime electrically in a molecular solid-state device, thus opening new fields of application to this general theoretical concept. Our observation of the MIR was possible for a number of underlying physical reasons. In the first place, we must recall that we inject electrons with high energies, which allow us to explore a transport regime beyond the one mapped with devices operating close to the Fermi level, such as conventional organic field-effect transistors. In the second place, our model system Au/fullerene represents a weakly interacting interface with relatively low interfacial disorder, which leads to the appropriate energy resolution required to observe the effective NDR. Moreover, LUMO and LUMO + 1 conductive levels are sufficiently separated and the electron-vibration coupling, which may lead to significant level broadening^35^, is not too large.

### Manipulation of the NDR

**Fig. 4:**
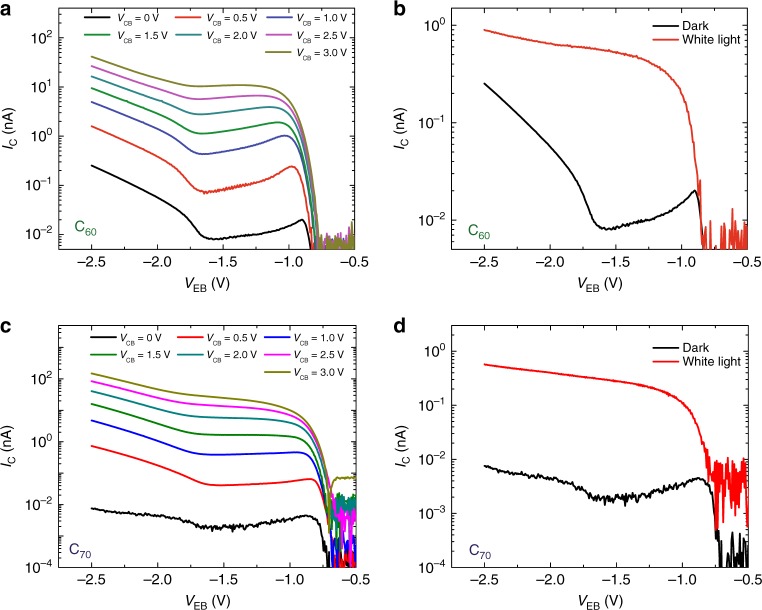
Manipulation of the negative differential resistance. **a** Dependence of the direct hot-electron current, *I*_C_, of a C_60_ hot-electron transistor on *V*_EB_ for different collector-base bias, *V*_CB_, at 180 K. **b** Hot-electron current *I*_C_ of C_60_ based device for *V*_CB_ = 0 V at 180 K in dark (solid black line) and under white-light irradiation (7.5 mW cm^−2^ illuminating an area of 1 cm^2^) (red solid line). **c** *I*_C_ of a C_70_ based hot-electron transistor on *V*_EB_ for different collector-base bias, *V*_CB_, at 180 K. **d** Hot-electron current *I*_C_ of a C_70_ based device for *V*_CB_ = 0 V at 180 K in dark (solid black line) and under white-light irradiation (7.5 mW cm^−2^ illuminating an area of 1 cm^2^) (red solid line)

The effective NDR arising from the MIR can be further manipulated by other external stimuli such as a collector-base voltage and light irradiation (see Fig. 4). In the first case, a positive voltage bias *V*_CB_ > 0 V can enhance the built-in potential inside the organic semiconductor, which in turn facilitates electron transport through this material. This extra potential leads to a larger current density, which progressively drives the system out of the MIR (see Fig. 4a, c) [see Supplementary Figs. 9–13 for data at other temperatures]. In the second case, the additional energy from the incident white-light spectrum (with power density of 7.5 mW cm^−2^) provides access to several high-energy transport levels such as LUMO + 1. This generated photocurrent in the C_60_ and C_70_ serves as an alternative parallel channel that overrides the effective NDR (see Fig. 4b, d) [the data at different temperatures with light irradiation is shown in Supplementary Figs. 14, 15b]. The manipulation of the NDR coming from the MIR is possible for both sets of fullerene samples.

## Discussion

In conclusion, by making use of the hot-electron injection technique in a three terminal solid-state device, we are able to actively access the MIR for electronic transport through an organic semiconductor thin-film. This regime, notoriously complex to demonstrate unambiguously in organic thin films, opens the possibility to both novel charge injection physics in molecular semiconductors as well as functionality in electronic circuiting such as NDR. Moreover, we are not only able to observe the MIR, but our three-terminal device allows us to manipulate and tune it by temperature, electric field, and light.

## Methods

### Device fabrication

All the devices described in this work were fabricated in situ in an ultra high vacuum (UHV) multi chamber evaporator (base pressure < 10^−9^ mbar) with a shadow mask system. The emitter is a 12 nm-thick aluminium contact, 99.95% purity (Lesker), which was thermally evaporated in an effusion cell with a rate of 0.6 Å s^−1^. A crucible of pyrolytic boron nitride (PBN) was used. The Al_2_O_3_ tunnel junction was made by plasma oxidizing the aluminium contact, first for two minutes at low power (1200 V and 10 mA at 0.1 mbar) and then for three minutes at high power (1200 V and 50 mA at 0.1 mbar). A 10 nm-thick gold base (99.95% purity, Lesker) was e-beam evaporated from a vitreous-coated graphite-based crucible, and used as base contact. The evaporation rate was 1.0 Å s^−1^. C_60_ (and C_70_) triple-sublimed quality (99.9%, Sigma Aldrich), was thermally evaporated in a quartz-based crucible with a rate of 0.1 Å s^−1^. 200 nm and 100 nm thick C_60_ and C_70_ films were used, respectively. Finally, a 12 nm-thick aluminium top electrode was again thermally evaporated. A two-step deposition (2 nm at 0.1 Å s^−1^ and 10 nm at 0.6 Å s^−1^) was followed in order to minimize the damage to the organic film.

The sample size is 10 × 10 mm^2^, and six devices were produced in every sample. The measurements were reproducible between devices in the same evaporation.

### Electrical characterization

Electrical characterization was performed under high vacuum (base pressure 5 × 10^−5^ mbar) in a variable-temperature probe-station (Lakeshore). A Keithley 4200 semiconductor analyser system was used to record *I*-*V* curves.

## Supplementary information

Supplementary Information

## Data Availability

The datasets generated and analyzed during the current study are available from the corresponding author on reasonable request.

## Code availability

The code employed during the current study is available from the corresponding author on reasonable request.
