# Supplementary material for: Tuning the charge flow between Marcus regimes in an organic thin-film device
Source: Nat Commun. 2019 May 7;10:2089. doi: 10.1038/s41467-019-10114-2 (PMC6504872; doi:10.1038/s41467-019-10114-2)
Supplement: Supplementary file 1 — Supplementary Information [file 41467_2019_10114_MOESM1_ESM.pdf]

Supplementary Information

Tuning the charge flow between Marcus regimes in an organic thin-film device

A. Atxabal, T. Arnold, S. Parui, S. Hutsch, E. Zuccatti, R. Llopis, M. Cinchetti, F. Casanova, F. Ortmann, L.E. Hueso

Correspondence to: frank.ortmann@tu-dresden.de; l.hueso@nanogune.eu

Supplementary Figures

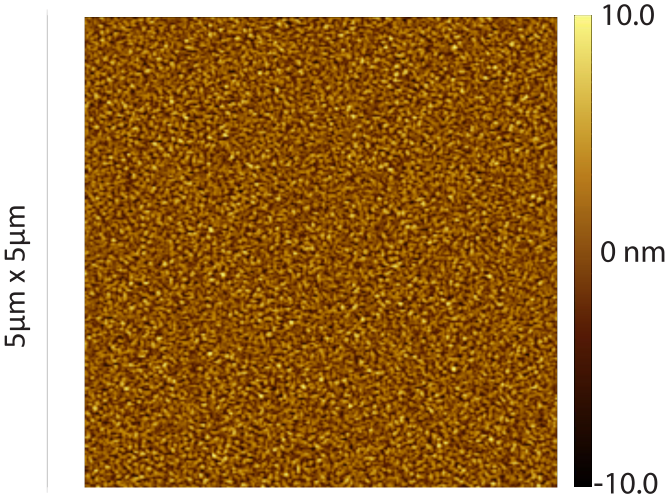

Supplementary Figure 1| Atomic force microscopy (AFM) picture of 200nm of C_{60}. AFM picture of 200nm of C_{60} thermally evaporated on 10 nm-thick gold thin film with a rate of 0.1 Å s^{-1}. The root mean squared roughness of the film is 1.6 ± 0.1 nm.

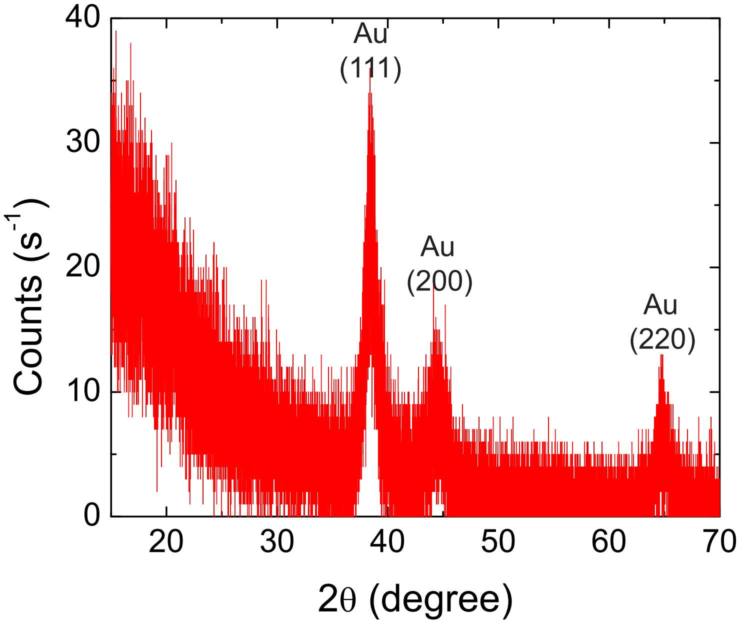

Supplementary Figure 2| X-ray diffraction measurement of 200 nm-thick C_{60} film on 10 nm-thick gold thin film. Three peaks are observed in the diffraction patterned, which corresponds to Au (111), (200) and (220) [1,2]. No peak has been observed for C_{60}, which ensures its amorphous morphology.

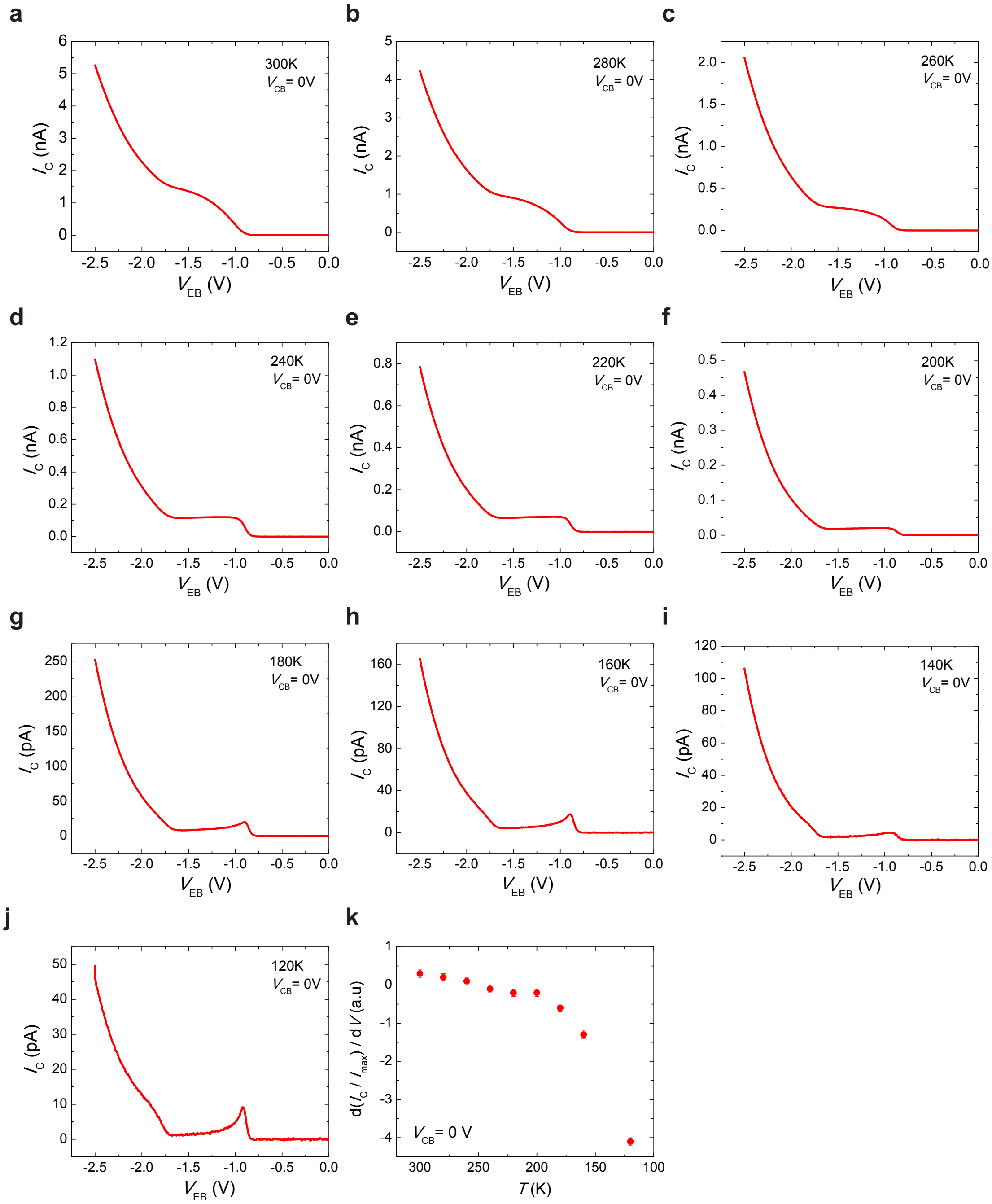

Supplementary Figure 3| Temperature dependence of collector current I_C for negative emitter-base bias $V_{EB} < 0$ V in a Au/C_{60} hot-electron transistor. Collector current I_C for negative emitter-base bias $V_{EB} < 0$ V at temperatures from 300 K to 120 K for a hot-electron transistor with an Au/C_{60} interface. Energetic electrons are injected and detected with V_{CB} = 0V into the lowest molecular orbital (LUMO) and next higher excited conductive molecular level (LUMO+1) of C_{60}. The increase of I_C between LUMO and LUMO+1 at high temperatures (**a-c**) is observed to converge into a plateau at 240 K (**d-f**). At lower temperatures, below 200 K, a decrease in the I_C

is observed between LUMO and LUMO+1 (**g-j**). Figure **k** corresponds to the maximum change in the I_C with temperature where effective negative differential resistance NDR is observed. Curves were normalised and the derivative calculated yielding the minimum point of the effective NDR. This minimum is represented for temperature from 300 K to 120 K with V_{CB}= 0 V. Error bars are s.e.m.

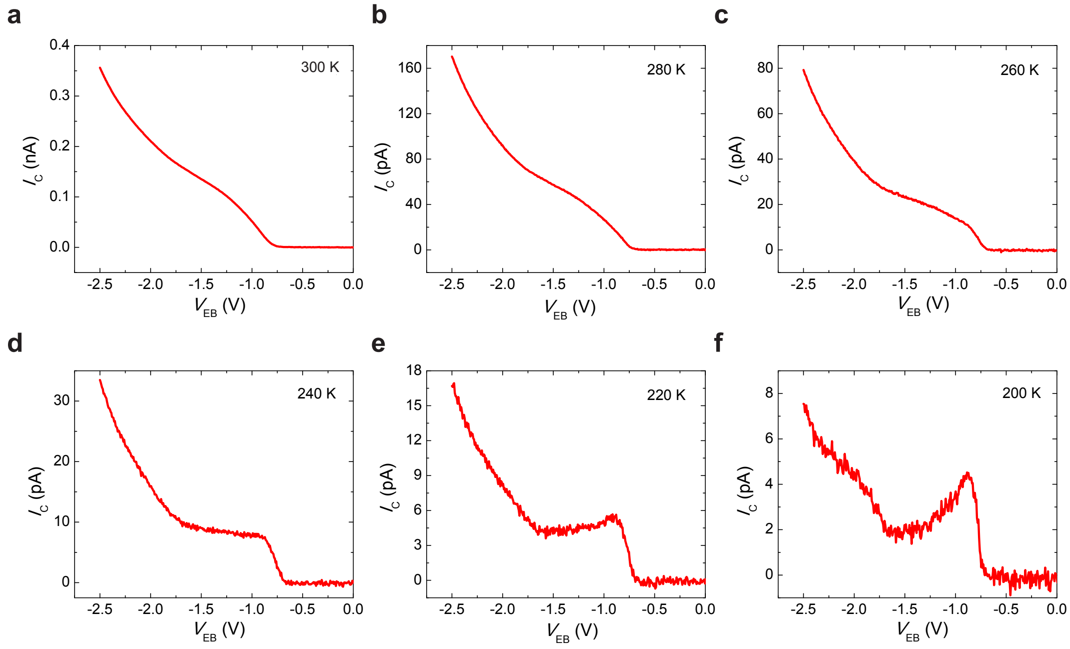

Supplementary Figure 4| Temperature dependence of collector current I_C for negative emitter-base bias $V_{EB} < 0$ V in a Au/C70 hot-electron transistor. Collector current I_C for negative emitter-base bias $V_{EB} < 0$ V at temperatures from 300 K to 200 K for a hot-electron transistor with Au/C_{70} interface. Energetic electrons are injected and detected with $V_{CB} = 0$V into the lowest molecular orbital (LUMO) and next higher excited conductive molecular level (LUMO+1) of C_{70}. The increase of I_C between LUMO and LUMO+1 at high temperatures (**a**-**c**) is observed to converge into a plateau at 240 K (**d**). At lower temperatures, below 220 K, a decrease in the I_C is observed between LUMO and LUMO+1 **(e-f)**

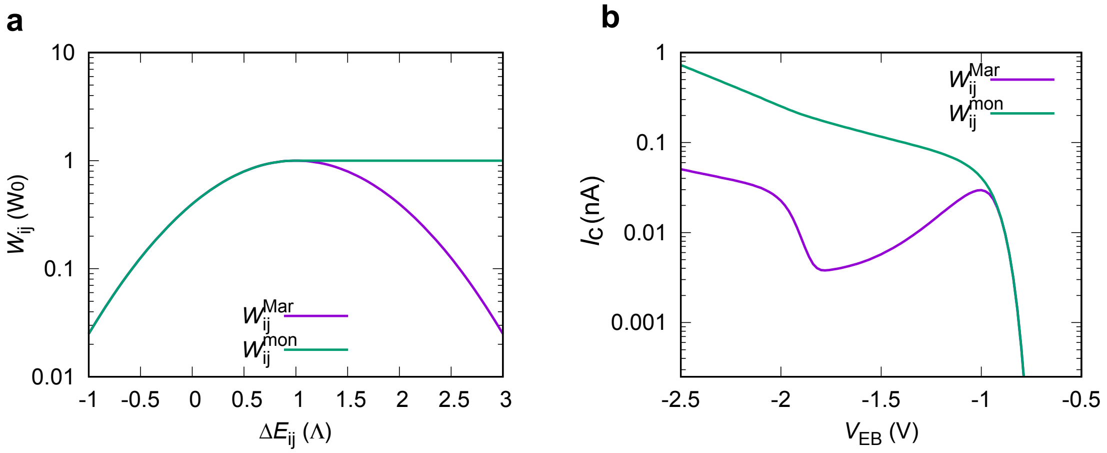

Supplementary Figure 5| Influence of the electron-transfer (hopping) rate on the MIR. a, Non-monotonous Marcus rate W_{ij}^{Mar} and monotonous rate W_{ij}^{mon} as a function of the energy difference ΔE_{ij} between initial and final transport states (in units of the reorganisation energy Λ) for temperature T = 120 K. **b**, Comparison of different rates regarding the direct hot-electron current I_{c} for vanishing base-collector bias V_{CB} = 0 and low temperature T = 120 K as a function of negative emitter-base bias V_{EB}. The MIR observed in the experiment cannot be modelled by using monotonous rates. See Supplementary Note 1 for a detailed discussion.

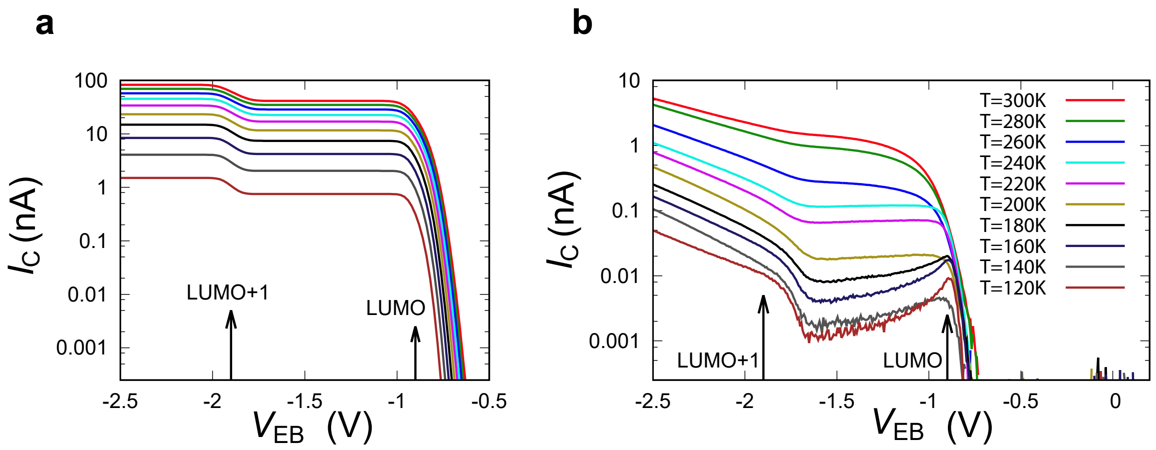

Supplementary Figure 6| Device characteristics in absence of a tunnel barrier. a, Temperature dependence of the collector current, I_C, versus emitter base bias, V_{EB}, in the case when no tunneling barrier is present (assuming constant, i.e. energy-independent tunneling probability) and when no inelastic scattering in the metal occurs. This model describes a biased metal-molecular layer-metal junction. In this case, the hot electrons originating from all states close to or below the Fermi level of the emitter electrode at -eV_{EB}, which match in energy to the LUMO and LUMO+1 levels, are transferred into the organic semiconductor. When the emitter Fermi level is raised above the energy of the respective molecular level, a step is observed in the collector current but no negative differential resistance (NDR) is observed between the LUMO and LUMO+1 levels of the C_{60}. **b**, Experimental temperature dependence of I_C versus V_{EB} when the charges that arrive to the metal/organic semiconductor interface are hot. NDR is observed below 180 K between LUMO and LUMO+1.

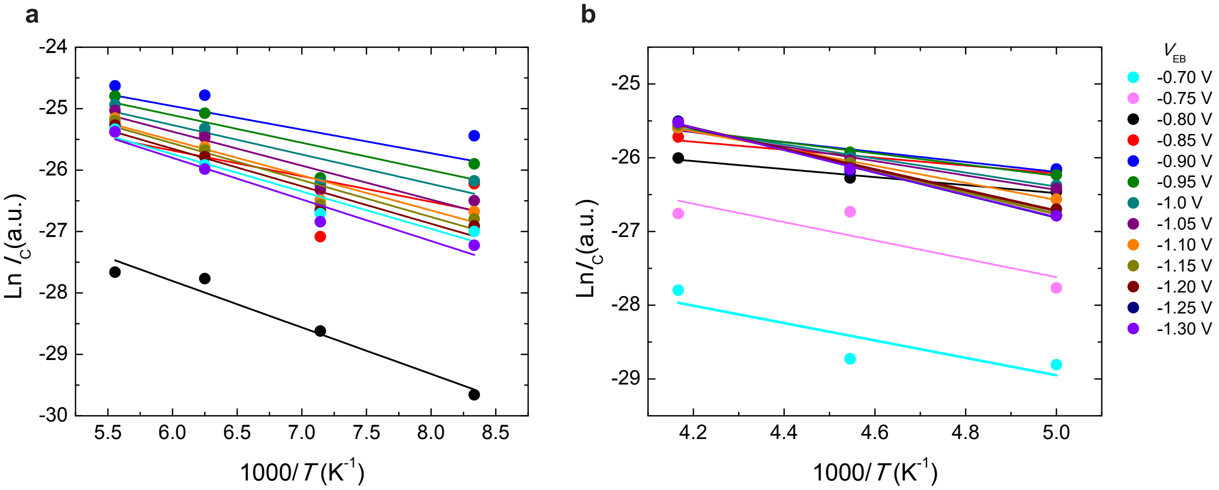

Supplementary Figure 7| Arrhenius plot. **a**, Arrhenius plot of I_C of C_{60} based hot-electron transistor measured at $V_{CB} = 0$ (colour points) from 180 K to 120 K in the emitter-base-bias range $-0.85\text{ V} \geq V_{EB} \geq -1.30\text{ V}$. The solid lines are fits to the Arrhenius equation. **b**, Arrhenius plot of I_C of C_{70} based i-MOS device measured at $V_{CB} = 0$ (colour points) from 240 K to 200 K in the emitter-base-bias range $-0.70\text{ V} \geq V_{EB} \geq -1.30\text{ V}$. The solid lines are fits to the Arrhenius equation.

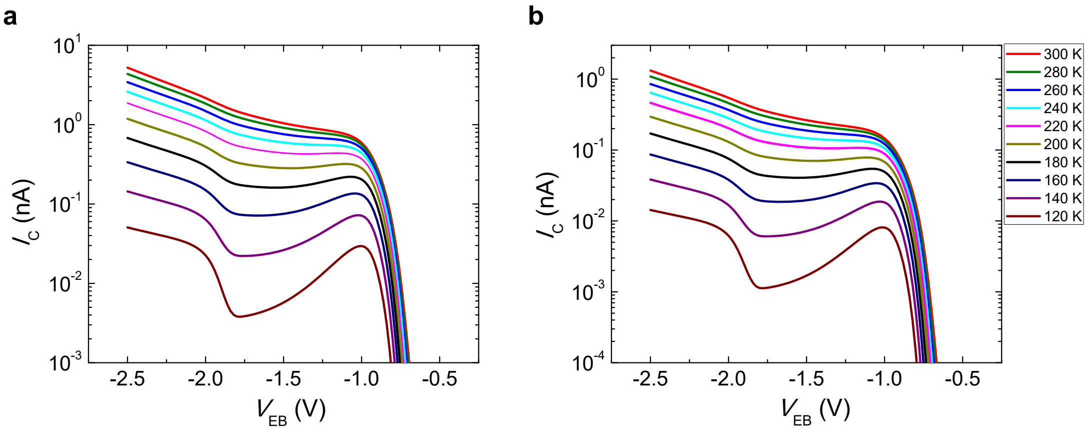

Supplementary Figure 8| Simulation of the length dependence of the hot-electron current in C_{60}. **a**, The layer thickness of the simulated C_{60} film corresponds to an experimental thickness of 200 nm (for more information, see Supplementary Note 1). **b**, The layer thickness of the simulated C_{60} film corresponds to a thickness of 400 nm in experiment.

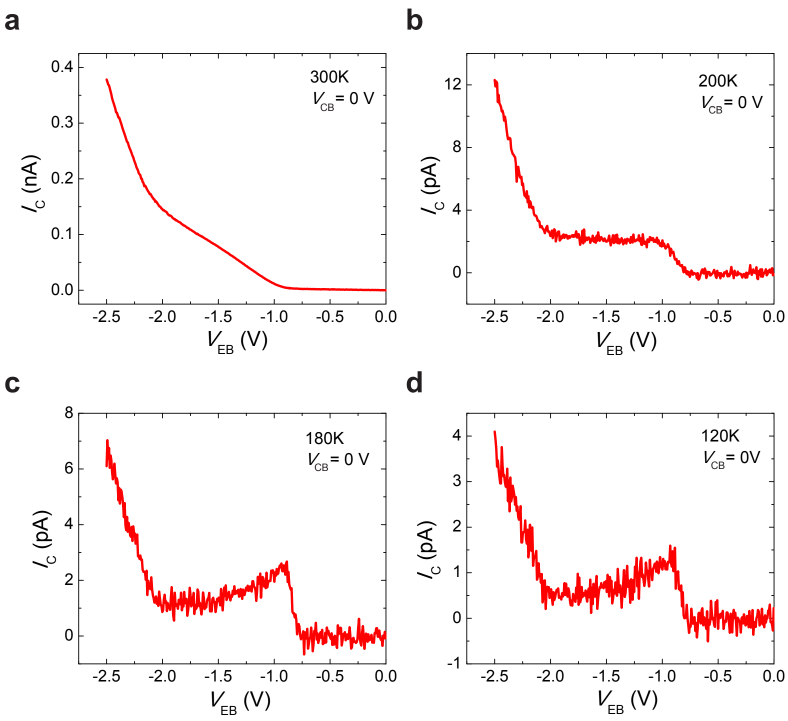

Supplementary Figure 9| Temperature dependence of collector current I_C for negative emitter-base bias $V_{EB} < 0$ V of Cu/C_{60} based hot-electron transistor. Collector current I_C for negative emitter-base bias $V_{EB} < 0$ V at temperatures from 300 K to 120 K for a hot-electron transistor with Cu/C_{60} interface. Energetic electrons are injected and detected with V_{CB} = 0V into the lowest molecular orbital (LUMO) and next higher excited conductive molecular level (LUMO+1) of C_{60}. The increase of I_C between LUMO and LUMO+1 at high temperatures (**a**) is observed to converge into a plateau at 240 K (**b**). At lower temperatures, below 180 K, a decrease in the I_C is observed between LUMO and LUMO+1 (**c**-**d**).

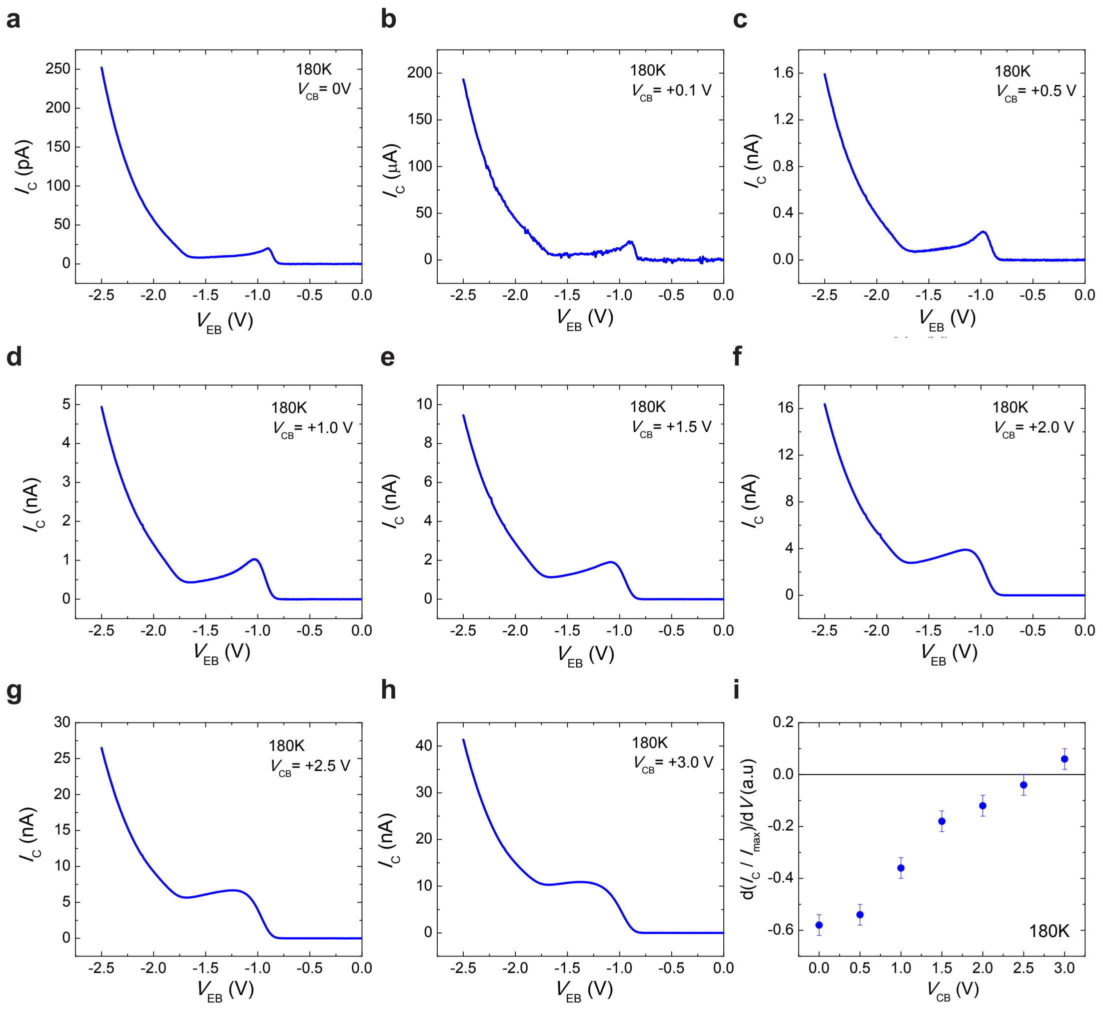

Supplementary Figure 10| Positive collector-base bias, V_{CB}, dependence of collector current I_C for negative emitter-base bias $V_{EB} < 0$ V at 180 K of a Au/C_{60} based hot-electron transistor. Energetic electrons are injected with $V_{CB} \geq 0$V into the lowest molecular orbital (LUMO) and next higher excited conductive molecular level (LUMO+1) of C_{60}. The lowering of the current observed between LUMO and LUMO+1 when no external V_{CB} bias is applied (**a**), gradually vanishes when higher positive V_{CB} is applied to the system (**b-i**). Figure **j** corresponds to the maximum change in the I_C at V_{EB} where effective negative differential resistance NDR is observed. The curves were normalised and the derivative respect to V_{EB} calculated yielding the minimum point of the effective NDR. This minimum is represented for positive V_{CB} at 180 K. Error bars are s.e.m.

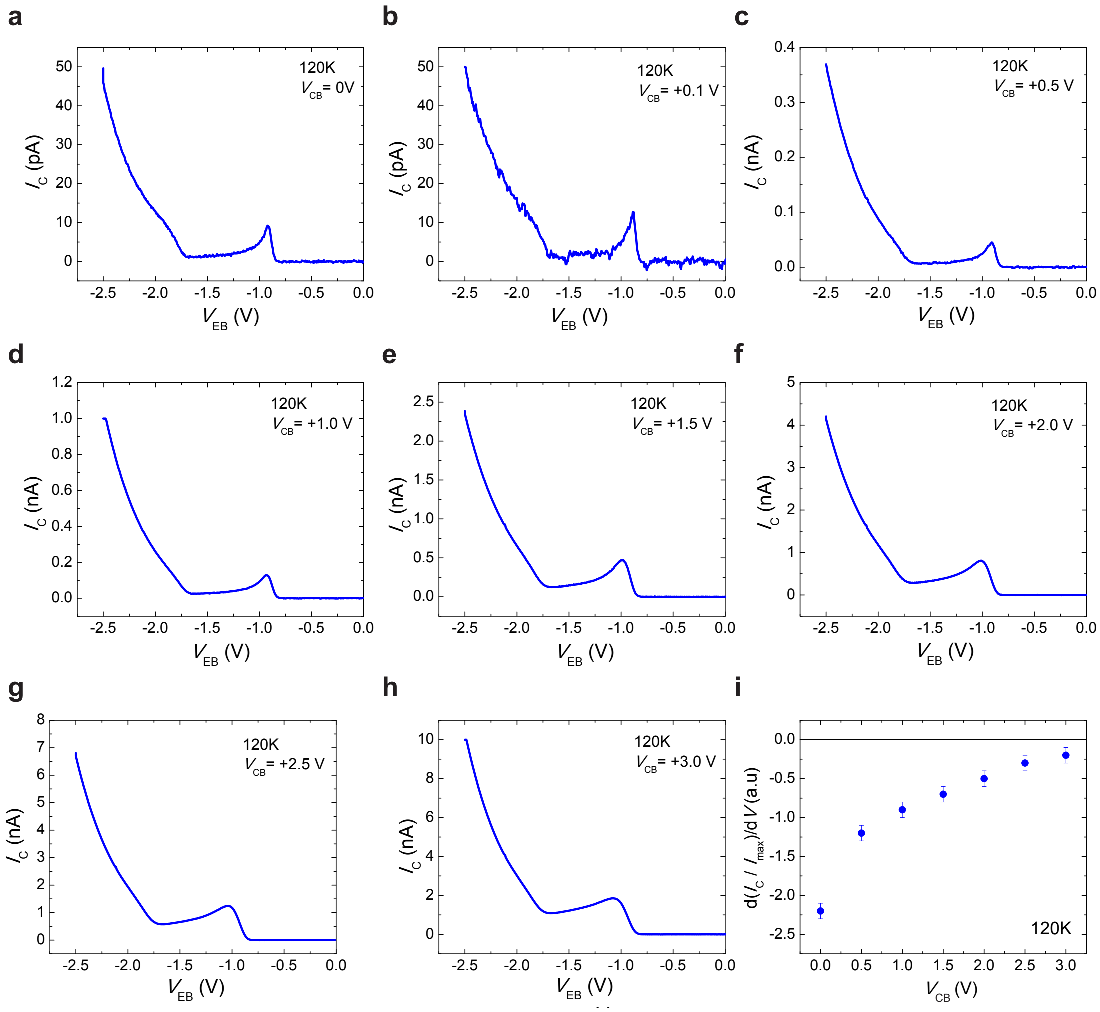

Supplementary Figure 11| Positive collector-base bias, V_{CB}, dependence of collector current I_C for negative emitter-base bias $V_{EB} < 0$ V at 120 K in a Au/C60 based hot-electron transistor. Energetic electrons are injected with $V_{BC} \geq 0$V into the lowest molecular orbital (LUMO) and next higher excited conductive molecular levels (LUMO+1) of C_{60}. The lowering of the current observed between LUMO and LUMO+1 when no external V_{BC} bias is applied (**a**), gradually becomes weaker when higher positive V_{BC} is applied to the system (**b-i**). Figure **j** corresponds to the maximum change in the I_C at V_{EB} where negative differential resistance NDR is observed. The curves were normalised and the derivative with respect to V_{EB} calculated yielding the minimum point of the NDR. This minimum is represented for positive V_{BC} at 120 K. Error bars are s.e.m.

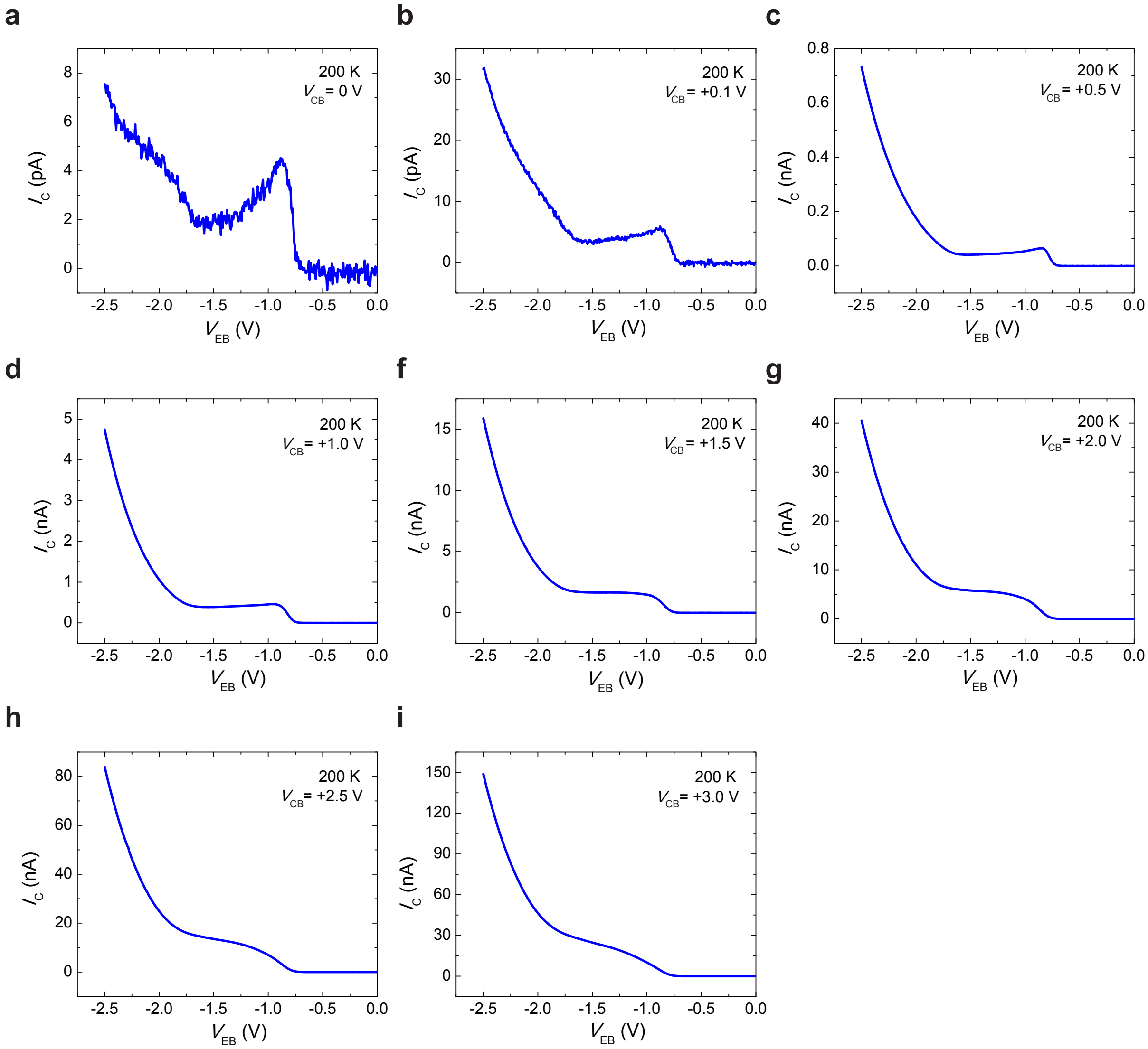

Supplementary Figure 12| Positive collector-base bias, V_{CB}, dependence of collector current I_C for negative emitter-base bias $V_{EB} < 0$ V at 200 K in a Au/C_{70} based hot-electron transistor. Energetic electrons are injected with $V_{CB} \geq 0$V into the lowest molecular orbital (LUMO) and next higher excited conductive molecular level (LUMO+1) of C_{70}. The lowering of the current observed between LUMO and LUMO+1 when no external V_{CB} bias is applied (**a**), gradually vanishes when higher positive V_{BC} is applied to the system (**b-i**).

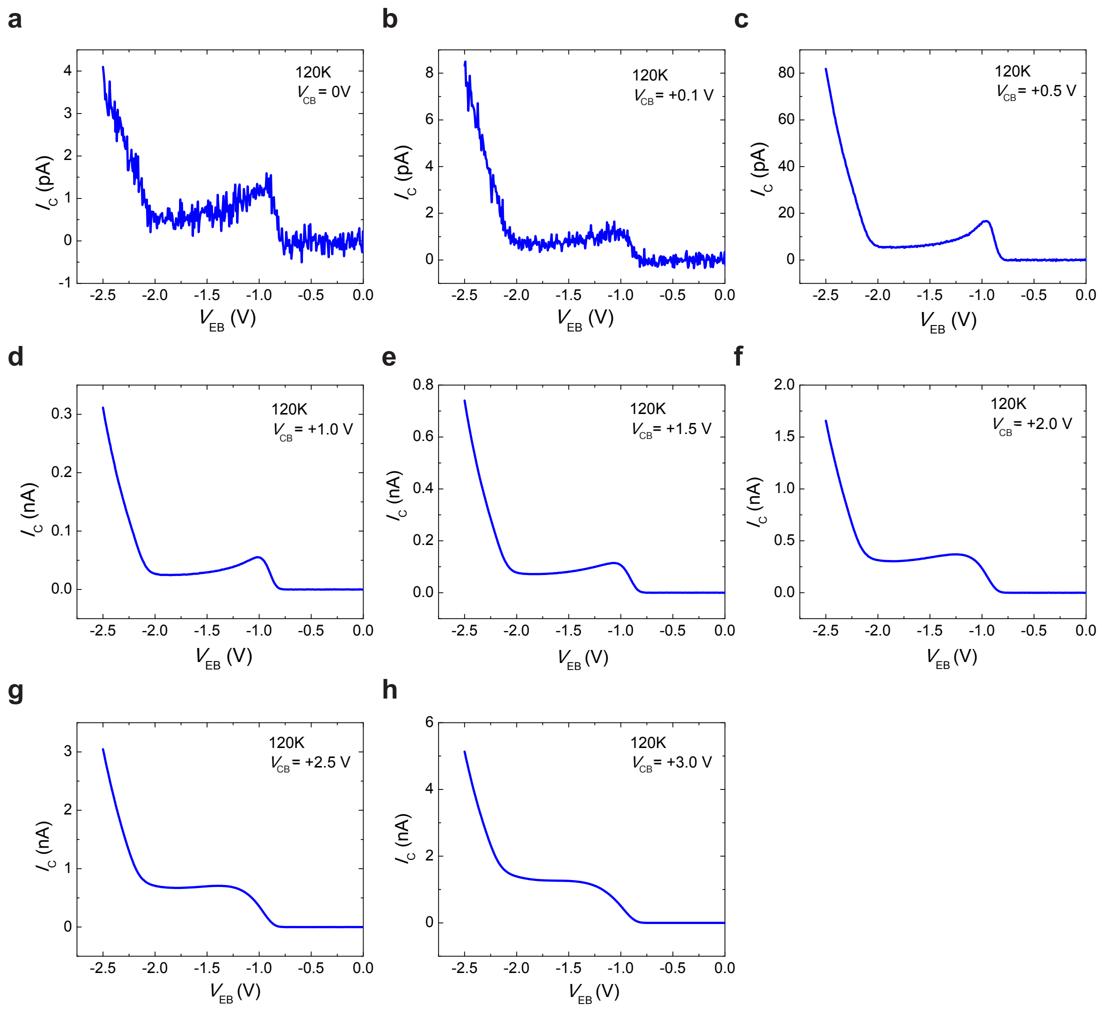

Supplementary Figure 13| Collector current I_C for negative emitter-base bias $V_{EB} < 0$ V at 120 K in a Cu/C_{60} based hot-electron transistor. Energetic electrons are injected with $V_{CB} \geq 0$V into the lowest molecular orbital (LUMO) and next higher excited conductive molecular level (LUMO+1) of C_{60}. The lowering of the current observed between LUMO and LUMO+1 when no external V_{CB} bias is applied (**a**), gradually vanishes when higher positive V_{BC} is applied to the system (**b-h**).

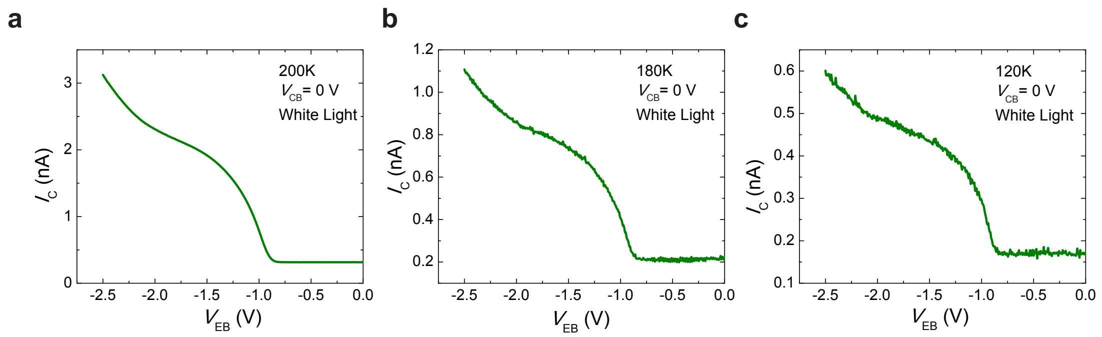

Supplementary Figure 14| Temperature dependence under light irradiation. Collector current I_C for negative emitter-base bias $V_{EB} < 0$ V at **a**, 200 K, **b**, 180 K and **c**, 120 K and under white light irradiation. Energetic electrons are injected with $V_{CB} = 0$V into the lowest molecular orbital (LUMO) and next higher excited conductive molecular level (LUMO+1) of C_{60}.

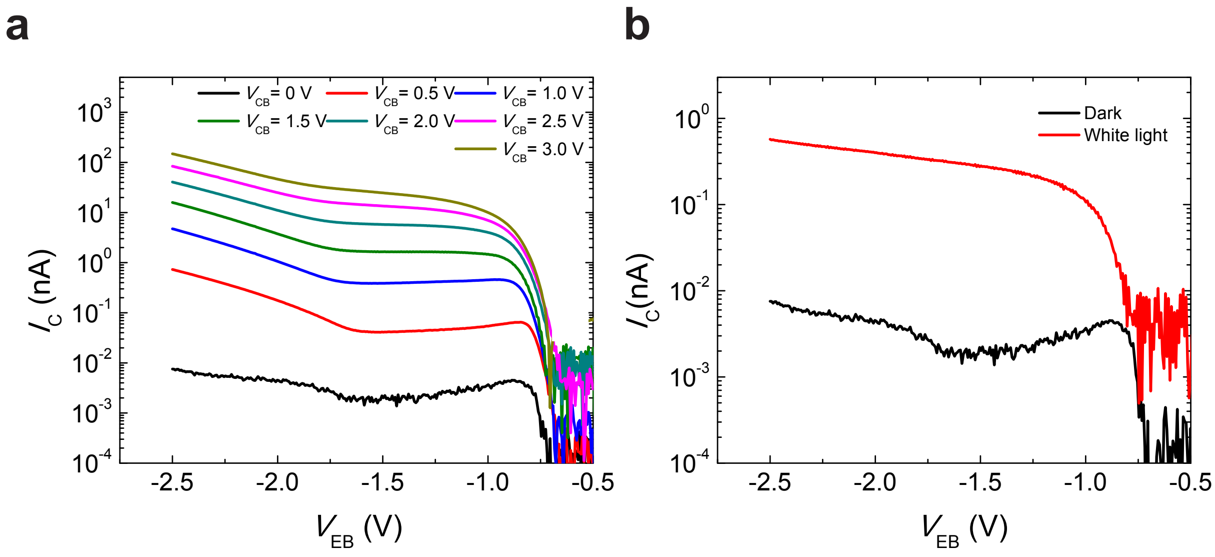

Supplementary Figure 15| Manipulation of the negative differential resistance of C_{70} hot-electron transistor. a, Dependence of the direct hot-electron current, I_C, on V_{EB} for different collector-base bias, V_{CB}, at 200K. **b**, Hot-electron current I_C for V_{CB} = 0 V in dark (solid black line) and under white-light irradiation (7.5 mW cm^{-2} illuminating an area of 1 cm^2) (red solid line).

Supplementary Tables

Supplementary Table 1| Activation energy extracted from Supplementary Figure 18a.

V_{EB} (V)	E_A (meV)
-0.80	65 ± 9
-0.85	40 ± 30
-0.90	30 ± 30
-0.95	40 ± 20
-1.0	40 ± 20
-1.05	47 ± 9
-1.10	49 ± 9
-1.15	52 ± 9
-1.20	53 ± 9
-1.25	53 ± 9
-1.30	59 ± 9

Supplementary Table 2| Compilation of simulation parameters. *b* is a parameter of the tunneling kernel describing the characteristics of the tunnelling barrier [3].

Parameter	C_{60}	C_{70}
ε_r	4.4	4.4
$\gamma_{C_{xx},C_{xx}}$	25 meV	25 meV
$\gamma_{C_{xx},Au}$	2.5 meV	2.5 meV
$\Lambda_{C_{xx},C_{xx}}$	110 meV	114 meV
$\Lambda_{C_{xx},Au}$	55 meV	57 meV
Δ_1^{LUMO}	0.83 eV	0.78 eV
Δ_2^{LUMO}	0.73 eV	0.58 eV
Δ_1^{LUMO+1}	1.83 eV	1.78 eV
Δ_2^{LUMO+1}	1.73 eV	1.58 eV
a	10 Å	11.6 Å
$\tilde{a}$	4 Å	4.6 Å
L	20 nm	20 nm
α	$10/a$	$10/a$
υ	0.01 ps^{-1}	0.01 ps^{-1}
p_i^{hot}	0.01	0.01
b	10	10
l_0	417 nm	417 nm
σ_{if}	25 meV	25 meV
σ_{bulk}	25 meV	65 meV

Supplementary Notes

Supplementary Note 1|Theoretical Modelling

Summary of temperature dependent transport approach: To model the hot-electron device, our approach describes the tunnelling through the emitter-base barrier, the elastic and inelastic scattering in the base metal, hot-electron injection into the organic semiconductor and hopping from molecule to molecule in the organic semiconductor[3].

Firstly, the hopping transport is modelled via a semi-classical master equation[4]

$$\frac{\partial p_i}{\partial t} = -\sum_{j \neq i} W_{ij} p_i \left(1 - p_j\right) + \sum_{j \neq i} W_{ji} p_j \left(1 - p_i\right) \tag{1}$$

where p_i is the electron occupation of a molecular orbital and W_{ij} is the hopping rate between localized transport states (indexed by i and j). Equation (S1) is solved for the stationary case. The hopping rates in Eq. (S1) are obtained from Fermi's golden rule in which the electronic coupling is treated as the perturbation parameter. Following Marcus, in presence of electron-vibration coupling, it evaluates to the well-known Marcus hopping rate [5]

$$W_{ij}^{\mathrm{Mar}} = \frac{2\pi}{\hbar} \frac{\gamma^2 \exp[-2\alpha(|\boldsymbol{r}_i - \boldsymbol{r}_j| - a)]}{\sqrt{2\pi\Lambda k_B T}} \exp\left[-\frac{\left(E_i - E_j - \Lambda\right)^2}{4\Lambda k_B T}\right] \tag{2}$$

where Λ is the reduced reorganisation energy[6] of the charge transfer event between the fullerene orbitals (see Supplementary Tab. 2 for all parameters), γ is the nearest neighbour transfer integral, a is the nearest-neighbor hopping distance and E_i are the site energies of the transport states. These energies result from the molecular transport states, which are modified by the built-in potential (the work function difference of the base and collector electrode), the image charge potential, the space charge potential, disorder and the polarization potential. The level offset Δ_1 (Δ_2) represent the energetic barrier between a transport level at the metal-organic interface and the Fermi energy in the base (collecting electrode) in absence of the image charge, space charge and polarisation potential and without disorder. The built-in field can be directly calculated from $E_{\mathrm{built-in}} = \frac{\Delta_1 - \Delta_2}{d}$, where d is the thickness of the fullerene film.

These simulations are performed on a regular lattice for which we choose a simple cubic structure. This choice also allows to obtain and include the space charge contribution of the charge carriers, which is realized as a laterally averaged quantity (solving a 1D Poisson equation) and hence depends on the coordinate along the transport direction of the device. Disorder and morphology effects of the experimental samples are included in a simple energetic disorder model. The disorder is assumed to follow a Gaussian distribution with standard deviation σ_{if} at the interface and σ_{bulk} in the bulk of the organic film which is in general assumed to be different (parameters σ_{if} and σ_{bulk} in Supplementary Table 2).

Here, we model two transport levels, called LUMO and LUMO+1 to remind their molecular origin. A relaxation rate ν between the levels

$$\frac{\partial p_i^{LUMO}}{\partial t} = -\frac{\partial p_i^{LUMO+1}}{\partial t} = \nu p_i^{LUMO+1}(1 - p_i^{LUMO}) \tag{3}$$

is assumed. The results of our calculations however do not depend on the particular choice of ν.

In case of injection from the metal into the organic semiconductor or extraction from the organic film (into the metal), the delocalised metal states are taken as initial (or final) states with a lower electronic coupling $\gamma_{\mathrm{C_{60},Au}}$ between molecular and metal states. Therefore, Eq. (S2) can also be used with some minor modifications. In the case that a metallic state is involved, the reorganization energy in Eq. (S2) is just the molecular reduced relaxation energy of C_{60} ($\Lambda_{\mathrm{C_{60},Au}}$). In addition, extraction from the thin film into the metal is not subject to energetic constraints since electrons can freely exit due to the finite density of states in the metal for all relevant energies. The distance between nearest neighbor molecular and metal states is estimated to be $\tilde{a}$. Electron injection from hot (i.e. energetically high-lying) metallic states in the base with an occupation probability p_i^{hot} of hot electrons represents the main injection mechanism since thermal electrons are prevented to enter the organic film due to the very high energy barriers.

In the second modelling step, the semi-classical master-equation-based hopping approach allows to calculate the electrical current $I_{\mathrm{BC}}(\tilde{E})$ for hot-electron injection at energy $\tilde{E}$. The hot-electron current

$$I_{\mathrm{c}} = \int_0^{-eV_{EB}} dE\ t(U,E) \int_{E_F}^{E} d\tilde{E}\, s(E,\tilde{E}) I_{\mathrm{BC}}(\tilde{E}) \tag{4}$$

includes the tunnelling and scattering probabilities, $t(U,E)$ and $s(E,\tilde{E})$, respectively, for the tunnelling through the emitter-base barrier at energy E and the scattering in the base to energy $\tilde{E}$. $U = -eV_{EB}$ is the energy of the electrons at the Fermi level of the emitter electrode. For the temperature dependence, the scattering in the base is of high importance. The central quantity describing the scattering is the scattering kernel $s(E,\tilde{E})$, which gives the yield of the hot electrons that have tunnelled through the emitter-base barrier at energy E and are then available for injection into the organic semiconductor at energy $\tilde{E}$. The hot electrons can scatter elastically at the boundaries of the base, but can also scatter inelastically. We consider two types of inelastic scattering processes either by losing their energy gradually (in average steps of ε) describing mainly the electron-electron scattering or by being thermalised in the base, for example, by Auger processes (in the latter case electrons would need to overcome the barrier to enter the organic semiconductor when thermalised in the base). If the electrons survive thermalisation and enter the organic semiconductor at energy $\tilde{E}$, they have lost the energy $\Delta E = E - \tilde{E}$ by multiple inelastic scattering events (in steps of ε). The probability of thermalisation between two events, increases with the inelastic mean free path $\lambda(E)$ between two events, which is for gold[7].

$$\lambda(E) = \frac{177 eV^2 nm}{E^2} + 0.054 \frac{nm}{\sqrt{eV}} \sqrt{E} \tag{5}$$

at room temperature. The longer the inelastic mean free path, the higher the chance that the electrons thermalise. In general, we assume a typical power-law-temperature dependence for the inelastic mean free path

$$\lambda(E,T) = \frac{177eV^2nm}{E^2(T/T_0)^\alpha} + 0.054\frac{nm}{\sqrt{eV}(T/T_0)^\alpha}\sqrt{E} \tag{6}$$

that describes that inelastic scattering (of losing energy ε) becomes more likely with larger T. The reference temperature T_0 is the room temperature for which Eq. (S5) is recovered.
We aim for a continuous description, in which the sum over all events of $\lambda(E,T)$ becomes an integral over the inelastic mean free paths until an energy $\tilde{E}$

$$l(E,\tilde{E},T) \propto \frac{1}{eV}\int_{\tilde{E}}^{E} dE'\,\lambda(E',T) \tag{7}$$

Next, we stress that the scattering kernel is proportional to the probability of survival of a hot electron in the scattering processes to reach energy $\tilde{E}$

$$s(E,\tilde{E},T) \propto e^{-l(E,\tilde{E},T)/l_0} \tag{8}$$

and the inelastic mean free path

$$s(E,\tilde{E},T) \propto \lambda(\tilde{E},T) \tag{9}$$

because the number of injection attempts into the organic semiconductor increases with the inelastic mean free path as a hot electron will then remain at energy $\tilde{E}$ for a longer time. As a result, the scattering kernel reads

$$s(E,\tilde{E},T) = s_0 e^{-l(E,\tilde{E},T)/l_0}\lambda(\tilde{E},T) \tag{10}$$

with s_0 and l_0 being constants. With a typical quadratic power law ($\alpha = 2$) in the temperature dependence, we find excellent agreement with experimental results. With increasing temperature, the total travelled path $l(E,\tilde{E},T)$ and its associated residence time in the base for given energies E and $\tilde{E}$ shortens. Therefore, more hot electrons (which are not relaxed to the Fermi level) are available at energy $\tilde{E}$. When $-eV_{\mathrm{EB}}$ is much larger than the transport level in the organic semiconductor, the Marcus Inverted Region is active. In contrast, for high temperature, the hot electron can lose a considerable amount of energy without dropping from the scattering path thus contributing to injection into the organic semiconductor and the hot-electron current I_{c}. The temperature therefore destroys the Marcus Inverted Region.
For the simulations, we use the parameters for C_{60} and C_{70} from Supplementary Tab. 2. In the simulations, we use a smaller sample length than in our experiments. In Supplementary Tab. 2, L is the length of the system in transport direction, which is one order of magnitude smaller than the experimental one for numerical convenience. The built-in field is scaled accordingly to have the same magnitude as in the experiments. We have tested that the results are independent of this length provided that the built-in field due to the different work functions of the electrodes is held constant.

Additional insights into the Marcus Inverted Region (MIR): In order to model charge transport and the MIR, the Marcus rate is an essential ingredient. While its derivation follows from Fermi's golden rule arguments and does not require additional justification, it is insightful to compare this rate to other popular but simplified (or less justified) rates for which the hopping probability increases monotonically with the energy gain $\Delta E_{ij} = E_i - E_j$ of the hopping electron. A well-known example would be the Miller-Abrahams rate[8].

In the Marcus rate, the hopping probability is *non-monotonous,* i.e. it decreases when the energy gain ΔE_{ij} surpasses the reorganisation energy. Indeed, the electron transfer from a metallic level in the base at an energy $\tilde{E} \gg E_i$ (with i being a molecular state in which electrons are injected) is suppressed. This suppression is reflected in the reduction of current, i.e. the establishment of the MIR.

To illustrate this effect further, we compare two transfer rates. We define a rate that is monotonous but otherwise similar to the Marcus expression, i.e. it uses the same prefactor $W_0 = \frac{2\pi}{\hbar}\frac{\gamma^2 \exp[-2\alpha(|\boldsymbol{r}_i - \boldsymbol{r}_j| - a)]}{\sqrt{2\pi\Lambda k_B T}}$. The modification consists in making it monotonous in the energy gain ΔE_{ij} of the hopping electron:

$$W_{ij}^{\mathrm{mon}} = \begin{cases} \frac{2\pi}{\hbar}\frac{\gamma^2 \exp[-2\alpha(|\boldsymbol{r}_i - \boldsymbol{r}_j| - a)]}{\sqrt{2\pi\Lambda k_B T}} \exp\left[-\frac{(E_i - E_j - \Lambda)^2}{4\Lambda k_B T}\right] & \text{if } E_i - E_j \leq \Lambda \\ \frac{2\pi}{\hbar}\frac{\gamma^2 \exp[-2\alpha(|\boldsymbol{r}_i - \boldsymbol{r}_j| - a)]}{\sqrt{2\pi\Lambda k_B T}} & \text{if } E_i - E_j > \Lambda \end{cases} \quad (11)$$

We compare it with the standard Marcus rate from Eq. (S2) in Supplementary Fig. 5a. Both rates equally increase for energy gains below the reorganisation energy. The Marcus rate decreases for higher energy gains and is hence a *non-monotonous* function of ΔE_{ij}, while the modified monotonous form W_{ij}^{mon} becomes constant.

We employ the different rates in the hot-electron transistor simulations and compare the result in Supplementary Fig. 5b. As opposed to the Marcus rate, for which the MIR is observed, the use of the monotonous rate W_{ij}^{mon} cannot reproduce the negative differential resistance between the LUMO- and LUMO+1-levels even at low temperatures. This is a generic result that applies for all rates with a monotonous energy dependence.

Parameters. There are two sorts of parameters used in our theoretical model. The overwhelming majority of parameters are material parameters, which are either simulated or known from literature. These parameters specify the material in the simulations. There are very few fit parameters, which are explained below. We included a list of all parameter values in the Supplementary Table 2.

The reorganization energy is based on the molecular reduced relaxation energy (Λ) of C_{60}, which we obtained from DFT calculations of electron-vibration coupling constants for the molecules (we

focus for the present discussion on the LUMO states, which are probed in Fig. 3a at about -0.9V). From the simulations of the molecular vibrations, we obtain Λ by first calculating the partial reorganization energies $\Lambda_i = S_i \hbar\omega_i$ for all molecular vibrational modes of the C_{60} (and C_{70}) species (S_i are the Huang-Rhys factors) and by summing these contributions over the low-frequency vibrations (semi-classical modes). According to prior work (Ref.[6]), the intramolecular high-frequency modes must be excluded from the semi-classical broadening (and hence Λ) that enters the exponential form of the Marcus expression. The concept of the reduced reorganisation energy leads to a lower broadening in the Marcus rate as compared to the traditionally used total reorganization energy. The DFT calculations employed use the B3LYP functional (with the Grimme DFT-D3 dispersion correction) and the 6-311G** basis set. Finally, the reorganization energy used for the organic bulk transport is twice the relaxation energy.

E_i and E_j are the electronic site energies of the C_{60} states (e.g. the LUMO or LUMO+1) or, if the index j labels a metallic site at the surface, its energy (e.g. a state at the Fermi energy or a hot electron level). These level energies in the organic material are determined self-consistently from the Poisson equation (including space charges, possible additional bias V_{CB}, image charge potential, possible disorder and polarization potential) and the initial Schottky barrier heights (Δ_1 and Δ_2) at the electrodes. The latter are chosen in consistency to our measurements. The image charge potential is estimated from the distance between the Au surface and C_{60} and the dielectric constant of C_{60} of 4.4. Also, the difference between LUMO and LUMO+1 is well established in literature.

The quantities r_i and r_j indicate positions of the electronic states for the hopping model, which are defined by the lattice model for the structure of the organic semiconductor. The transfer integrals γ between neighbouring C_{60} sites are set to a constant. The value is estimated as average value from *ab initio* calculations. For further distant hops the transfer integrals are exponentially suppressed, and this distance-dependence is described by the factor containing the inverse localization length α, which can be modeled considering the transfer integral distributions for neighbouring molecular localized states calculated for the varying distances. In conclusion, we have undertaken a large effort to fix all these material parameters when unknown (by *ab initio* simulations) to avoid that they enter as fitting parameters.

Regarding the energy broadening, this is found to have a minor effect at the interface for fullerenes. If this was not the case, the hot-electron onset in the normal regime would not be so sharp at V_{EB}~-0.75 V but would be more strongly broadened by disorder. Energy offset and the effect of level alignment (if present) manifest in the position of the injection barrier, i.e. the transport level relative to the metal Fermi level, and therefore in the onset of the hot-electron current. Our observations and main conclusions are not affected by the value of the injection barrier. In the theoretical model, as mentioned above, we have chosen an offset that is in consistency to our measurements.

In addition, there is another parameter which is not accessible, namely the transfer integral γ_{C60-Au} for the injection from the metal into C_{60}, which affects the absolute value of the current. It is an empirical parameter and determines the prefactor of the hot-electron current (the same holds for the chosen bulk disorder). The transfer integral is very difficult to model and as we do not aim at predicting the exact magnitude of the current (which actually depends on unknown experimental details, e.g. the tunnelling barrier) it is used as a fit parameter.

We would like to emphasize that all our main findings are qualitatively unaffected by the chosen values for these few free parameters. Importantly, the choice of these parameters does not affect the occurrence of the Marcus Inverted Region in the simulations.

Supplementary References

1. Luo, X. *et al.* Remarkably enhanced red–NIR broad spectral absorption via gold nanoparticles: applications for organic photosensitive diodes. *Nanoscale* **7,** 14422–14433 (2015).
2. Tan, Z. *et al.* Facile deposition of gold nanoparticles on C_{60} microcrystals with unique shapes. *J. Nanoparticle Res.* **15,** 2029–2039 (2013).
3. Arnold, T., Atxabal, A., Parui, S., Hueso, L. E. & Ortmann, F. Hot Electrons and Hot Spins at Metal-Organic Interfaces. *Adv. Funct. Mater.* **20,** 1706105–15 (2018).
4. Van Der Holst, J. J. M. *et al.* Modeling and analysis of the three-dimensional current density in sandwich-type single-carrier devices of disordered organic semiconductors. *Phys. Rev. B - Condens. Matter Mater. Phys.* **79,** 085203-11 (2009).
5. Marcus, R. A. Chemical and electochemical electron-transfer theory. *Annu. Rev. Phys. Chem.* **15,** 155–196 (1964).
6. Vandewal, K. *et al.* Absorption tails of donor:C_{60} blends provide insight into thermally activated charge-transfer processes and polaron relaxation. *J. Am. Chem. Soc.* **139,** 1699–1704 (2017).
7. Seah, M. & Dench, W. Quantitative Electron Spectroscopy of Surfaces. *Surf. Interface Anal.* **1,** 2–11 (1979).
8. Miller, A. & Abrahams, E. Impurity Conduction at Low Concentrations. *Phys. Rev.* **120,** 745–755 (1960).
